# Usefulness of Procalcitonin Levels for Predicting the Microbiological Orientation in Patients with Sepsis

**DOI:** 10.3390/jpm14020208

**Published:** 2024-02-15

**Authors:** Natalia Fernanda Pascual Gómez, María del Pilar Sanz Martín, María Auxiliadora Semiglia Chong, Nelly Daniela Zurita Cruz, Rosa Méndez Hernández, Iñigo Guerra Molina, Iñigo García Sanz, Angels Figuerola Tejerina, Fernando Ramasco Rueda

**Affiliations:** 1 Department of Clinical Analysis, La Princesa University Hospital, Diego de León 62, 28006 Madrid, Spain; mariadelpilar.sanz@salud.madrid.org; 2 Physiology and Pathophysiology Teaching Unit, Faculty of Pharmacy, Complutense University of Madrid, 28040 Madrid, Spain; 3 Department of Microbiology, La Princesa University Hospital, Diego de León 62, 28006 Madrid, Spain; auxisemig2@gmail.com (M.A.S.C.); nellydaniela.zurita@outlook.com (N.D.Z.C.); 4 Department of Anesthesiology and Surgical Intensive Care, La Princesa University Hospital, Diego de León 62, 28006 Madrid, Spain; rosamen2004@hotmail.com (R.M.H.); gorria66@gmail.com (F.R.R.); 5 Department of Emergency, La Princesa University Hospital, Diego de León 62, 28006 Madrid, Spain; inigo.guerra@salud.madrid.org; 6 Department of Digestive and General Surgical, La Princesa University Hospital, Autónoma University of Madrid, Diego de León 62, 28006 Madrid, Spain; garciasanzinigo@hotmail.com; 7 Department of Preventive Medicine and Public Health, La Princesa University Hospital, Diego de León 62, 28006 Madrid, Spain; angels.figuerola@gmail.com

**Keywords:** biomarkers, sepsis code, procalcitonin, microbiology

## Abstract

The main objective of the study was to verify whether levels of procalcitonin (PCT) could guide us toward determining the type of bacteria causing the sepsis and to identify the discriminatory cut-off point in the first urgent laboratory test. This study is a single center retrospective analysis that includes 371 patients with a mean age of 71.7 ± 15.6 years who were diagnosed with sepsis or septic shock. The yield of blood cultures in demonstrating the causative microbiological agent was 24.3% (90), and it was 57, 1% (212) when evaluating all types of cultures. Statistically significant positive differences were observed in the mean value of the PCT between the group that obtained positive cultures and the group that did not (*p* < 0.0001). The AUC-ROC of PCT values as a guide to the causal bacteria type was 0.68 (95%CI: 0.57–0.78, *p* < 0.0021). The PCT value that showed the best diagnostic characteristics for identifying Gram-negative rods (GNR) as the causative agent in blood cultures was 2.1 ng/mL. The positive predictive value (PPV) was 78, 9% (66.3–88.1%). The AUC-ROC of the PCT values for sepsis diagnosis, with any positive culture that could be assessed, was 0.67 (95%CI: 0.63–0.73, *p* < 0.0001). The PCT value that showed the best diagnostic characteristic for predicting sepsis was 3.6 ng/mL.

## 1. Introduction

Sepsis diagnosis remains a clinical challenge. It has the potential to be disastrous. Without early detection and standardized care protocols for these patients, the mortality of this disease is very high. Sepsis performance improvement programs, which optimize organizational and educational processes to provide the best possible diagnosis and treatment in sepsis, have been shown to improve outcomes [1,2]. The Surviving Sepsis Campaign clinical guidelines [3] suggest the creation of such programs in different hospital centers.

The sepsis pathophysiological response involves different biochemical and immunochemical molecules that are expressed by different human tissues in molecular signaling pathways. The detection of these molecules, known as biomarkers, can be a very useful tool to help early diagnosis, predict the course of the disease, and evaluate the therapeutic efficacy [1]. Great efforts have been made in the search for the ideal biomarker for the diagnosis of sepsis. The reality is that there is no ideal biomarker. However, the combination of multiple biomarkers with clinical scales and their kinetics has proven to be very useful in supporting clinical diagnosis [4].

There have been several studies that compared different biomarkers for the diagnosis of sepsis. PCT and lactate have shown the greatest early diagnostic and prognostic capacity, taking into account the availability of both in hospital centers [5,6,7,8].

Microbiological cultures remain the gold standard for diagnosing infections. However, due to their low sensitivity and the intrinsic delay in obtaining results, efforts continue to be made to search for rapid biomarkers, together or in combination with clinical scales, that guide us with regard to the course of sepsis and orient us toward the responsible microbiological agents that can allow us to establish the earliest effective treatment [9]. Non-personalized empirical treatments are one of the main causes of an increasing percentage of multiresistant infections [10,11]. Efforts have been made to try to demonstrate the usefulness of biomarkers to predict the type of microbiological agent responsible for sepsis in order to apply the most effective therapy as soon as possible. However, there are several publications that warn about the limitations of these non-ideal biomarkers and that they can cause false elevations or false non-elevated levels [12]. This represents a field in which to continue research.

The usefulness of PCT as a predictor of the microbiological agent responsible for sepsis has not yet been clarified and is not fully accepted in the scientific community; however, it is frequently considered in evidence-based medicine in hospitals [13]. The main objective of this study was to verify whether there is an association between the serum levels of PCT and the type of bacterial causal agent and to establish a PCT value in the first blood analysis with the best compromise between the sensitivity and specificity to predict this.

The secondary objective was to compare the sepsis diagnostic capacity of the different biomarkers, identify the best one, and establish a diagnostic cut-off point for the first blood test regardless of the focus.

## 2. Materials and Methods

### 2.1. Study Population

This retrospective study analyzed all the patients who were activated with the Sepsis Code alert between January 2019 and December 2019 at the La Princesa University Hospital of Madrid, (Spain) according to the Guidelines on Surviving Sepsis campaign [3].

Exclusion criteria were patients who were not activated or who were deactivated because the infectious origin of the clinical condition was not confirmed.

### 2.2. Measurement of Blood Biomarkers and Microbiology Analysis

Upon admission, patients’ blood and plasma samples were collected to quantify the different biomarkers of inflammation and infection (PCT, C Reactive Protein (CRP), blood count), as well as those biomarkers that allow us to evaluate the specific organ involvement (basic biochemistry, renal profile, liver profile, and blood gas analysis that includes lactate). Measurement was made with flow cytometry and photometric, immunochemical, and amperometric assays (XN-1000 Sysmex, Kobe, Japan, cobas^®^ 6000 analyzer series Roche Diagnostics, Germany, GEM^®^ Premier™ 5000 Werfen, Bedford, MA, USA). These measurements were made as part of routine clinical practice.

Patients’ blood, urine, surgical, respiratory, or stool cultures, as appropriate, were collected from the corresponding clinical services. All samples were collected for the correct clinical care of the patients and were analyzed in the Clinical Analysis and Microbiological Laboratories of La Princesa University Hospital.

Blood samples were cultured in Bactec/Alert bottles (Becton Dickinson, Durham, NC, USA). Positive samples were rapidly identified, and antimicrobial susceptibility was tested using MicroScan (Beckman Coulter, Brea, CA, USA) and blood cultures using, at the same time, Accelerate-Pheno. All bacterial isolates were identified by analyzing the colony morphology, Gram staining, and using a MALDI-TOF spectrometer MALDI Biotyper^®^ (Bruker Daltonics, Billerica, MA, USA) which has become the reference method for the identification of microorganisms, allowing microbiology laboratories to identify microbes reliably, quickly, and cost-effectively. For the same patient, a microbiological identification assessed by the microbiology laboratory in blood culture was considered the gold standard. If this did not allow identification, the sample corresponding to the suspected focus was evaluated accordingly.

### 2.3. Study Variables

We collected demographic variables including the age, sex, type of patient, and service. The laboratories variables included PCT, CRP, the neutrophil/lymphocyte ratio (Ratio N/L), platelets (PLA), transaminases (ALT, AST, GGT), alkaline phosphatase (FAL), total bilirubin (BILT), creatinine (CRE), urea (URE), lactate (LACT), and the blood cell count (BCC) in the first urgent clinical blood analysis and the appropriate microbiological culture sample with the isolated microbiological agent and its identification.

The Sepsis Code alert was activated in patients with signs of organ dysfunction and clinical suspicion of infection. Activation of the Sepsis Code implies the start of diagnostic and treatment measures immediately, pending diagnostic confirmation, both in the extra-hospital and in-hospital settings. The activation of the Sepsis Code alert is carried out in the hospital computer system and is maintained for at least the first 72 h or until its existence is ruled out. To activate the alert, respiratory, inflammatory, tissue perfusion, hemodynamic, and organ dysfunction variables are taken into account. In addition to the clinical suspicion, the qSOFA scale is evaluated, and qSOFA ≥ 2 is the criteria for activating the alert. The alert is deactivated when the clinical suspicion of sepsis is ruled out.

### 2.4. Statistical Analysis

For the descriptive analysis of qualitative variables, frequencies were calculated, and Pearson’s χ^2^ test or Fisher’s exact nonparametric test was used for comparisons. For quantitative variables, the mean was calculated with its standard deviation (SD), and comparisons were made using Student’s *t*-test or the nonparametric Mann–Whitney U test.

To select the best discriminatory value of the PCT and other interesting biomarkers, for the diagnosis of sepsis and for the association with GNR, the area under the ROC curves (AUC) of the mean were calculated and compared. The sensitivity, specificity, positive predictive value (PPV), negative predictive value (NPV), positive likelihood ratio (PVR), negative likelihood ratio (NRV), and probability of correct diagnosis or correct microbiological association were calculated for PCT with a higher AUC. To identify the discriminatory PCT value that is associated with sepsis due to GNR, patients with blood cultures were chosen, since the bacterial load and its dissemination, depending on the focus, can significantly influence the biomarker values. Finally, using the selected PCT values as a cut-off for the test, we calculated the diagnostic properties and the Odds Ratio (OR).

Data analysis was carried out using the statistical software MedCalc for Windows version 22.013. Differences were considered statistically significant at *p* < 0.05.

## 3. Results

### 3.1. Descriptive Data

This study included 400 Sepsis Code alert activations. Of them, 372 corresponded to patients who were finally diagnosed with sepsis or septic shock. In one patient, it was not possible to perform the first urgent blood test; so, biomarker results could not be obtained, leaving 371 valid patients who met the criteria and from whom complete data were collected. The average age of the patients was 71.7 ± 15.5 years, and 63.6% (236) were male and 36.4% (135) female. The frequencies according to the corresponding services that activated the alert are shown in (Table 1). The emergency department activated the most Sepsis Code alerts.

The yield of blood cultures that demonstrated the causative microbiological agent in patients with sepsis was 24.3% (90) (blood cultures), and it was 57, 1% (212) evaluating all types of cultures (blood, urine, surgical, respiratory, and stool cultures).

**Figure 1:**
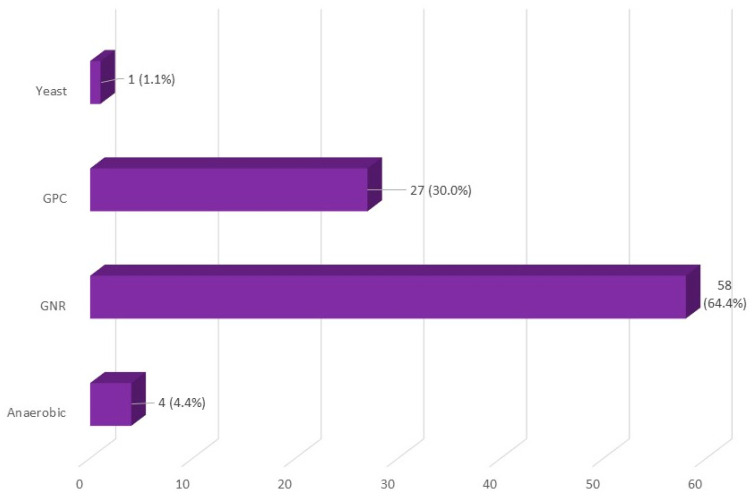
Type of microbiological agent isolation in positive blood cultures. GPC: Gram-positive cocci, GNR: Gram-negative rods.

**Figure 2:**
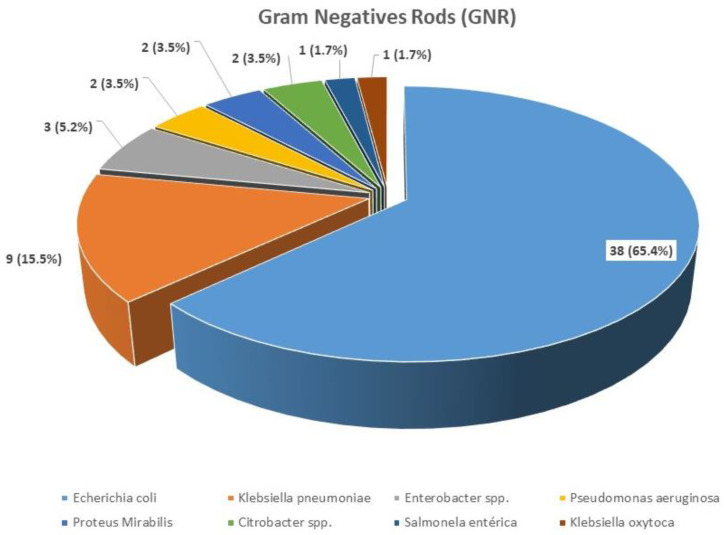
Type of GNR identification in positive blood cultures.

**Figure 3:**
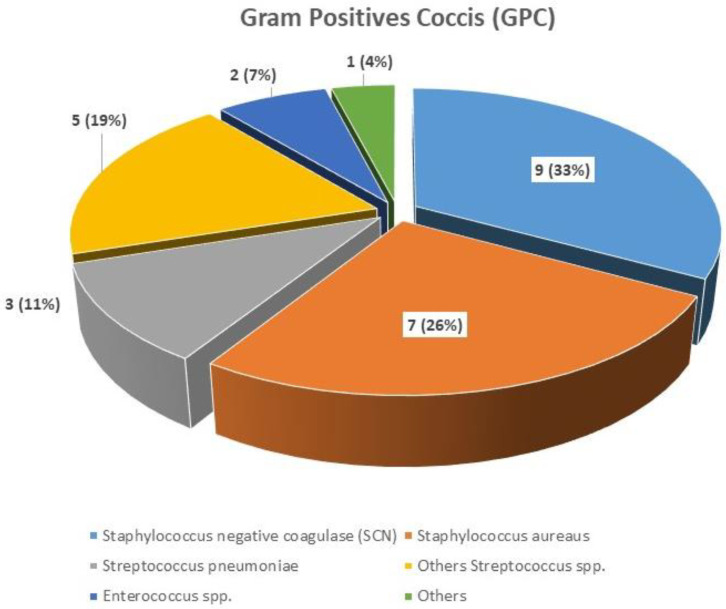
Type of GPC identification in positive blood cultures.

The frequency depending on the type of sample culture and the microbiological agent are shown in Table 2 and Figure 1, Figure 2 and Figure 3. *Escherichia coli* was the most frequent microbiological agent identification in positive blood cultures (63%).

### 3.2. Outcome Data (Primary Outcome)

**Figure 4:**
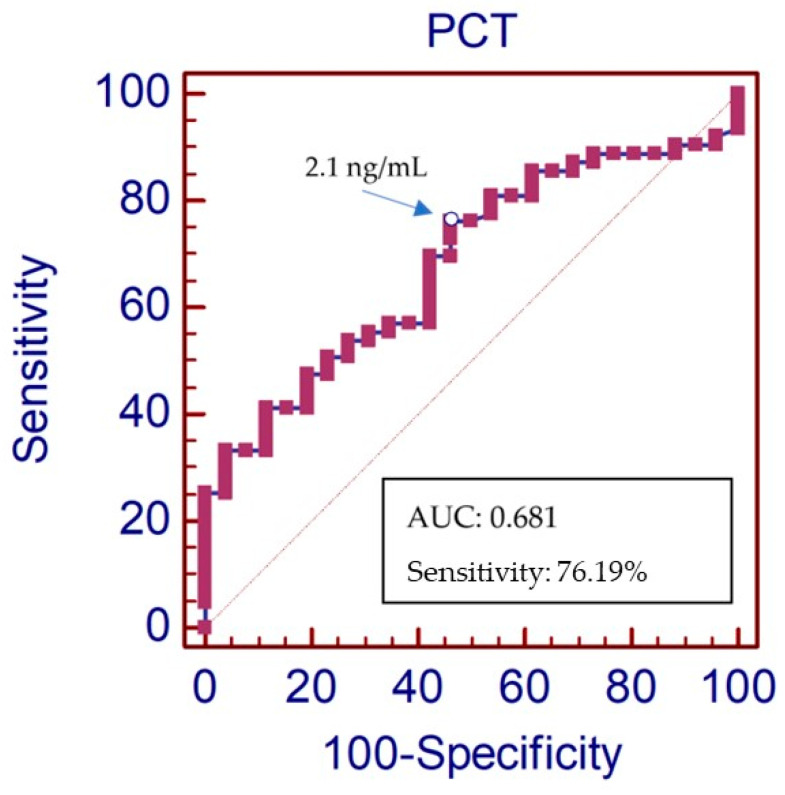
ROC curve of the microbiological orientation values of PCT in patients with sepsis with Gram-negative agents in blood cultures. The red diagonal dotted line represents a classifier that is no better than random guessing.

**Figure 5:**
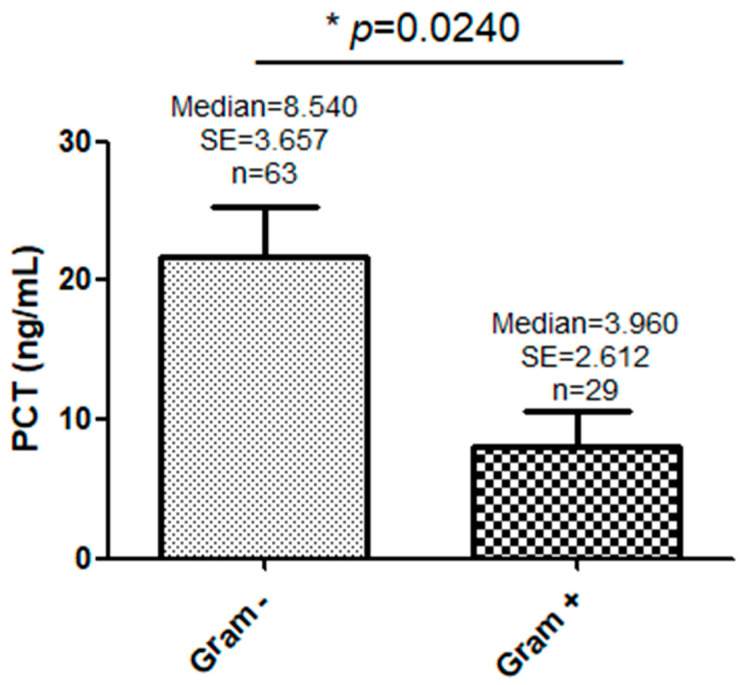
Comparisons of PCT concentrations and the type of microbiological bacterial agent isolation in blood cultures. * Statistically significant.

The area under the ROC curve (AUC-ROC) of PCT values as a guide to the causal bacteria type was 0.68 (95% CI: 0.57–0.78, *p* < 0.0021) (Figure 4). The PCT value that showed the best compromise in terms of the sensitivity and specificity for predicting the causal type of bacteria in blood cultures was 2.1 ng/mL. The positive predictive value (VPP) was 78.9% (95% CI: 66.3–88.1%). An analysis of the guide to the causal bacteria-type characteristics of PCT ≥ 2.1 ng/mL is described in detail in Table 3. With the characteristics described in Table 3, the probability that a patient with a PCT value ≥ 2.1 ng/mL (in the first blood analytical sample) actually had a GNR as the causal type of bacteria of the sepsis was 78,7%. The odds ratio (OR) for the causal GNR bacteria type in the group who had a PCT value ≥ 2.1 ng/mL was 3,2 (95% CI 1, 22–8, 38; *p* < 0.018). Comparisons of the PCT concentrations in the sepsis or septic shock patients with a positive blood culture and the type of microbiological agent isolation are shown in (Figure 5). Statistically positive differences (*p* = 0.0240) were observed between the medians of the group of patients with GNR isolation and the group of patients with GPC isolation (8.54 ng/mL and 3.96 ng/mL, respectively).

If we take into account all types of cultures, not just blood cultures, the cut-off point for the microbiological orientation to GNR changes to a PCT value ≥ 7.8 ng/mL, and the specificity increases to the detriment of the sensitivity (E: 75.0% (95% CI: 59.7–86.8%) S: 37.3% (95% CI: 28.6–46.7%) VPP: 80.0% (95% CI: 67.0–89.6%), with a non-significant OR.

### 3.3. Outcome Data (Secondary Outcome)

The mean value of the PCT in patients who were diagnosed with sepsis or septic shock with any positive culture evidence was 15.5 ng/mL (95% CI: 11.8–19.2), and the median was 3.8 ng/mL (95% CI: 2.1–6.0). Statistically positive differences were observed between the mean PCT and GGT in the groups that obtained positive cultures or not (*p* < 0.0001). Non-statistically significant differences were observed among the means of other sample biomarkers (CRE, GOT, GPT, URE, FAL, BILT, CRP, BCC, PLA, Ratio N/L, and lactate).

**Figure 6:**
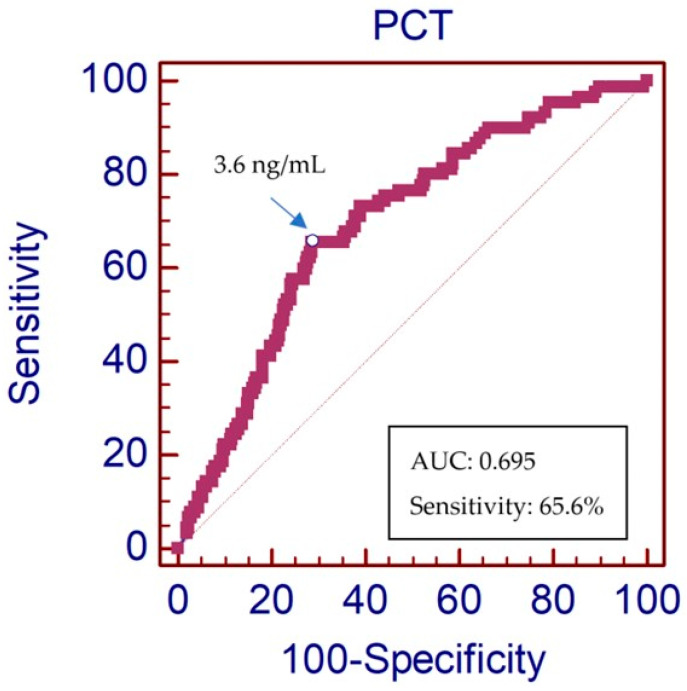
ROC curve of the diagnostics values of PCT in sepsis or septic shock patients with positive blood cultures. The red diagonal dotted line represents a classifier that is no better than random guessing.

The AUC-ROC of the PCT values for the sepsis diagnosis, with a positive blood culture, was 0.69 (95% CI: 0.64–0.74, *p* < 0.0001) (Figure 6).

The PCT value, which showed the best compromise between the sensitivity and specificity for predicting sepsis, with a positive blood culture, was 3.6 ng/mL. The sepsis prediction characteristics of a PCT ≥ 3.6 ng/mL are described in detail in Table 4. The OR of predicting sepsis or septic shock in the group who had a PCT value ≥ 3.6 ng/mL and positive blood culture was 4.7 (95% CI 2.8–7.8; *p* < 0.0001)

The AUC-ROC of the PCT values for sepsis diagnosis, with any positive culture that can be assessed, was 0.67 (95% CI: 0.63–0.73, *p* < 0.0001). As we can see, the cut-off point did not change when all types of cultures were taken into account, compared to only blood cultures. However, in this case, a high VPN prevailed, which was 84.8% (79.1–89.4%).

## 4. Discussion

Our results confirm the association between the serum levels of PCT and the type of causative microbiological agent, like GNR, with the results of the first urgent blood analysis. The value of PCT, with better compromise between the sensitivity and specificity in predicting the type of causative microbiological agent was 2.1 ng/L.

Most of the patients in our study were elderly people, as reflected by the average age (71.7 ± 15.5 years), with associated comorbidities, a true reflection of the population attended to in our health area. These characteristics indicate an increased probability of complicated infections, which are often the origin of sepsis or septic shock. It is precisely the elderly who present the physiological situation of immunosenescence (compared to other patients with a limited inflammatory reaction or more nonspecific clinical manifestations such as neonates or immunosuppressed patients), where the use of biomarkers of infection has experienced the greatest increase in urgent situations [14]. Inappropriate empiric antimicrobial therapy in patients over 65 years of age with sepsis was associated with a twofold reduction in 2-year survival [15].

Research has shown that the main stimulus for PCT secretion is bacterial endotoxin, and systemic infections cause an increase that is more marked than local infections [16]. This explains why the PCT cut-off point, taking into account all types of microbiological cultures, to orient toward the causal bacterial agent loses significance in the calculation of the OR compared to blood cultures.

As in other studies, microbiological documentation was obtained in 57.1% of patients with sepsis or septic shock, a figure comparable to the 60–70% previously described [15,17,18].

Other publications have found, like us, a higher frequency of bacterial infections due to GNR in the elderly population [19].

Other previous publications have also found higher PCT values associated with GNR, especially in bacteremia due to Enterobacteria. However, the cut-off points are different in the scientific community [20]. This reflects the need to study the cut-off points by age groups, foci, and the day of biomarker analysis in relation to the pathophysiological course of the disease.

Our results and the characteristics of PCT to guide the type of causative bacteria are similar to those previously reported in the literature. Leli et al. [21] reported, in a population with an age group similar to ours, a cut-off point for PCT of 1.6 nmol/L vs. 2.1 nmol/L in our study, with similar results in relation to the medians in the group of patients with sepsis caused by GNR vs. GPC (13.8 ng/mL and 2.1 ng/mL vs. 8.54 ng/mL and 3.96 ng/mL, respectively) and statistical significance.

Liu et al. [22] reported a study in which they compared the ability of PCT to the ability of CRP to determine the type of bacteria causing sepsis. In their study, they obtained better results from the ROC curve for a PCT of 0.73 (95% confidence interval 0.65–0.81) than a CRP of 0.52 (95% confidence interval 0.43–0.62), coinciding with the cut-off point with our study, 2.1 ng/mL.

The most widely described PCT and probability of sepsis reference values establish a range of 2 to 10 ng/L for a high probability of sepsis with a high risk of multiorgan failure. This is in line with our results that establish 3.6 ng/L as the cut-off point with the best sensitivity–specificity relationship. It must be taken into account that our cut-off point was established in patients who met the clinical criteria for the activation of the Sepsis Code alert and in the elderly population, where the majority had organ failure, which explains why our cut-off point is higher than other publications [23].

It was not possible to us to compare the diagnostic capacity of PCT and CRP with adequate population representation. The latter biomarker is not an urgent request, and most of the alerts were activated from the Emergency Service.

However, beyond comparing the diagnostic capacity, it seems more interesting to us, as long as there is the possibility of using both biomarkers, to highlight the complementary usefulness, especially when the diagnostic suspicion is due to another type of nonbacterial microbiological agent. In these cases, CRP can play an interesting role without forgetting that it is a positive acute phase reactant; so, its levels can increase up to 25% in cases of inflammation that are not necessarily of infectious origin [24].

Being able to find a single cut-off point regardless of the focus and whether or not there was ultimately microbiological identification is very useful, especially in urgent clinical decisions that require speed due to the illness of the patients. However, we must keep in mind that it is not an isolated or unique parameter of interpretation and that values less than 3.6 ng/mL PCT do not rule out the presence of sepsis or septic shock.

Although the Surviving Sepsis Campaign clinical guidelines [3] include a recommendation against the use of PCT as a diagnostic biomarker, in routine clinical practice, its use to support decision making is widespread and increasing.

The possibility of using objective tools such as analytical biomarkers in microbiological guidance provides an opportunity for therapeutic optimization and the fight against multimicrobiological resistance. For this, it is also very important to have annual reports on the sensitivities to the different bacteria in the hospital care area.

Without being the ideal biomarker of bacterial infection, PCT appears promising in terms of guidance on the type of causal bacterial agent.

In this sense, other authors such as Bassetti et al. have provided a decision algorithm for advance empirical treatment aimed at Gram-negative bacilli when PCT is >2 ng/mL, a cut-off point very similar to that obtained in our study [25].

We find it interesting for future studies to be able to extrapolate a PCT cut-off point that could serve as a guide in cases of sepsis and septic shock in which it is not possible to identify the causative bacteria in the blood culture. However, we find it important to distinguish the cut-off points, according to the location of the focus, and its spread can influence PCT values.

### Study Limitations

This study has several limitations regarding the usefulness of PCT for the prediction of the microbiological orientation in sepsis patients and according to the biases inherent to a retrospective study. The patients had different degrees of sepsis severity and dissemination of the microbiological agent, which influences the levels of the biomarkers. Furthermore, previous pathologies were not collected. Despite this, the results without individualizing subpopulations in first urgent care seem to be more useful, especially due to the immediacy of urgent care. These limitations, despite the possibilities that this study offers to elucidate the role of PCT in microbiological orientation, imply the need to carry out more prospective studies to determine the association of PCT in the prediction of the causative bacteria and its classification according to Gram stain, as well as the search for other tools that include biomarkers and improve the microbiological orientation of PCT.

## 5. Conclusions

Elevated PCT levels are associated with GNR. The PCT value in the first blood analytical sample, to orient toward the causal bacteria-type of sepsis being GNR, with the best compromise in sensitivity and specificity, was 2.1 ng/L. Patients with a high clinical suspicion of sepsis or septic shock with PCT > 2.1 ng/mL had a 78.7% probability that a GNR was the cause of infection. Therefore, PCT has been a good diagnostic predictive biomarker in patients with sepsis or septic shock, and the cut-off with the best compromise in sensitivity and specificity was 3.6 ng/L.

## Figures and Tables

**Table 1:** Demographics of the patients with an activated Sepsis Code alert.

Percentage (%)	Frequency (n)	Descriptive Data ^1^
63.6%	236	Male
36.4%	135	Female
30.2%	112	Patients admitted to ICU
44.7%	166	Emergency department
14.3%	53	Intensive care unit
10.2%	38	Anesthesia department
8.6%	32	Internal medicine and infectious department
6.2%	23	General and digestive surgery department
4.6%	17	Nephrology and hemodialysis department
3.5%	13	Urology department
2.2%	8	Neurology and neurosurgery department
1.6%	6	Medical oncology department
1.6%	6	Pneumology department
1.1%	4	Rheumatology department
0.8%	3	Hematology department
0.3%	1	Otorhinolaryngology department
0.3%	1	Cardiology department
**Median**	**Standard** **Deviation (SD)**	**Descriptive data ^2^**
71.7	±15.5	Age (years)
4.25	±2.24	SOFA
9.32	±11.6	Total days of stay in hospital

^1^ Sex, patients admitted to Intensive Care Unit (ICU), and departments that activated the Sepsis Code alert. ^2^ Age and clinical data.

**Table 2:** Frequency and percentage of the type of sample culture and the microbiological isolation.

Type of Sample Culture and the Microbiological Isolation		
Percentage (%)	Frequency (n)	Blood Culture (n = 371)
24.3%	90	Positive cultures
66.9%	248	Negative cultures
8.9%	33	Contaminated cultures
89%	80	Monobacterial isolation
11%	10	Polybacterial isolation
**Percentage** **(%)**	**Frequency** **(n)**	**Other samples evaluated microbiologically according to the focus (n = 281) ^1^**
18.5%	52	Urine culture
14.2%	40	Surgical culture
9.6%	27	Respiratory culture
1.1%	3	Stool culture
1.6%	6	Medical oncology department
1.6%	6	Pneumology department

^1^ Study of the patients with contaminated or negative blood cultures.

**Table 3:** Microbiological orientation characteristics of PCT ≥ 2.1 ng/mL.

IC 95%		Statistical Value	Microbiological Orientation Characteristics of PCT ≥ 2.1 ng/mL ^1^
Upper Limit	Lower Limit		
86.0%	63.8%	76.2%	Sensitivity
70.1%	29.9%	50.0%	Specificity
88.1%	66.3%	78.7%	Positive Predictive Value
66.1%	27.5%	46.4%	Negative Predictive Value
2.3%	1.0%	1.5%	Positive Likelihood Ratio
0.9%	0.3%	0.5%	Negative Likelihood Ratio

^1^ Study of the patients with positive blood cultures.

**Table 4:** Diagnostic characteristics of PCT ≥ 3.6 ng/mL.

IC 95%		Statistical Value	Diagnostic Characteristics of PCT ≥ 3.6 ng/mL ^1^
Upper Limit	Lower Limit		
75.3%	54.8%	65.6%	Sensitivity
76.5%	64.9%	71%	Specificity
54.0%	36.3%	45.0%	Positive Predictive Value
89.6%	79.4%	85.0%	Negative Predictive Value
2.9%	1.8%	1.5%	Positive Likelihood Ratio
0.7%	0.4%	0.5%	Negative Likelihood Ratio

^1^ Study of the patients with positive blood cultures.

## Data Availability

The raw data supporting the conclusions of this article will be made available by the corresponding author (nataliafernanda.pascual@salud.madrid.org) on request.
